# mSphere of Influence: Diagnosis of antifungal tolerance and persistence

**DOI:** 10.1128/msphere.00029-25

**Published:** 2025-07-28

**Authors:** Lei Chen

**Affiliations:** 1 Key Laboratory of Microbial Engineering Under the Educational Committee in Chongqing, Department of Microbiology, College of Basic Medical Sciences, Army Medical University 12525, Chongqing, China; Shenzhen Institute of Advanced Technology, Shenzhen, China

**Keywords:** antifungal tolerance, antifungal persistence, diagnosis, fungal single cell sequencing

## Abstract

Lei Chen works in the field of clinical diagnosis of antifungal tolerance and persistence. In this mSphere of Influence, he reflects on how the papers “Antifungal tolerance is a subpopulation effect distinct from resistance and is associated with persistent candidemia” by A. Rosenberg, I. V. Ene, M. Bibi, S. Zakin, et al. (Nat Commun 9:2470, 2018, https://doi.org/10.1038/s41467-018-04926-x) and “Macrophage internalization creates a multidrug-tolerant fungal persister reservoir and facilitates the emergence of drug resistance” by A. Arastehfar, F. Daneshnia, N. Cabrera, S. Penalva-Lopez, et al. (Nat Commun 14:1183, 2023, https://doi.org/10.1038/s41467-023-36882-6) impacted his work on the development of diagnostic methods for antifungal tolerance and persistence.

## COMMENTARY

Human pathogenic fungi affect approximately 1.7 billion people worldwide, causing conditions from superficial infections to life-threatening invasive diseases (1, 2). This public health challenge is exacerbated by limited therapeutic options, as only four antifungal classes are available to target invasive infections (3). Although these therapeutics are widely used against fungal infections, their efficacy is limited by significant challenges. In particular, overuse in clinical and environmental settings has led to widespread drug resistance, contributing to rising mortality rates from fungal diseases (4–6).

Antifungal resistance refers to the ability of fungi to survive and grow in the presence of antifungal drugs, mainly through genetic changes such as mutations or horizontal gene transfer (5, 7). It is usually quantified by measuring the minimum inhibitory concentration (MIC) of a particular antifungal drug, whereby resistant fungi are able to multiply and grow at concentrations of the drug that are fatal to other strains of the same species (5, 6). In addition to resistance, recent studies have highlighted the clinical importance of alternative modes of fungal survival during antifungal treatment, including antifungal tolerance and persistence (8). These phenomena are not associated with heritable mutations but arise from the heterogeneity of fungal cell populations, which enables fungi to survive antifungal challenges even when they are fully susceptible in standard microbiological assays (8–11).

To date, antifungal tolerance and persistence have been identified in all major pathogenic fungi, but our understanding of the determinants responsible for their development is still limited (10–13). Unlike antifungal resistance, antifungal tolerance and persistence are poorly characterized due to the lack of a comparable quantitative indicator (14). The identification of molecular and biological attributes that characterize these enigmatic cells is an important step for the clinical diagnosis of drug tolerance and persistence.

When considering the diagnosis of antifungal tolerance and persistence, I am inspired by the work of Rosenberg and colleagues (10) in the study “Antifungal tolerance is a subpopulation effect distinct from resistance and is associated with persistent candidemia,” in which the authors demonstrated an important link between antifungal tolerance and treatment outcomes and also began to unravel the mechanisms of tolerance of *Candida albicans* to the azole fluconazole. The work of Arastehfar and colleagues (11) in “Macrophage internalization creates a multidrug-tolerant fungal persister reservoir and facilitates the emergence of drug resistance” provided additional insights into mechanisms leading to antifungal tolerance. They found that internalization by macrophages induces tolerance and persistence during the fungal-host interaction.

Among the antifungal drugs, tolerance to the azoles (mainly tolerance of *C. albicans* to fluconazole) has been studied the most extensively (10, 15, 16). After exposure to fluconazole, all fungal cells in a population undergo a few rounds of division before growth arrest, but tolerant cells resume proliferation hours later, while non-tolerant cells take much longer or are unable to resume division (10). Using this distinctive phenotype, Rosenberg et al. (10) collected *C. albicans* strains from a variety of patients undergoing fluconazole-based treatment and revealed a significant correlation between drug tolerance and clinical treatment outcomes.

The data of clinical strains in this study highlighted the importance of antifungal drug tolerance, but they also provided useful insights into the mechanisms leading to tolerance. Fluconazole tolerance has been attributed to the heterogeneity of cells in a population (8). During drug treatment, levels of gene expression and metabolic rates vary within a population, leading to differences in responses to drug stress. Not surprisingly, Rosenberg et al. (10) determined that the factors underlying fluconazole tolerance are complex and can include altered rates of drug influx and efflux. However, they also determined that decreasing the activity of stress response pathways that regulate iron homeostasis and vesicular transport also had strong impacts on tolerance (10). Pharmacologic or genetic blockade of these pathways, including those involving calcineurin, Hsp90, and protein kinase C, improved fluconazole sensitivity (10). Inhibition of these pathways did not alter the MIC, suggesting that their effects are specific for fluconazole tolerance but not resistance (10). Importantly, the expression of genes related to these pathways may be useful as biomarkers for the diagnosis of fluconazole tolerance.

In bacteria, drug tolerance and persistence not only reduce the therapeutic efficacy but also accelerate the evolution of drug-resistant strains (17, 18). This phenomenon is particularly mediated by antimicrobial-induced oxidative stress, such as through elevated reactive oxygen species production, which enhances mutagenesis and consequently increases the probability of drug-resistant variant emergence (19). Recently, Arastehfar et al. (11) reported a similar mechanism in the human fungal pathogen *Candida glabrata*. In this pathogen, it has been well documented that echinocandin-resistant isolates emerge from genetically susceptible *C. glabrata* cells during infection. Based on this clinical observation, a hypothesis has been proposed that there are *C. glabrata* persister cells that survive fungicidal concentrations of echinocandins during infection, leading to the emergence of echinocandin-resistant isolates. Arastehfar et al. (11) found that internalization of *C. glabrata* by macrophages is associated with the formation of persisters that are highly tolerant to multiple antifungal drugs, and it also facilitates the emergence of echinocandin-resistant mutants (11). Disruption of genes involved in oxidative defense further promotes the emergence of drug-resistant mutants (11). This elegant study will inspire further basic (or “pre-clinical”) and clinical research that strives to elucidate host and fungal factors that support the development of tolerance and thus to identify additional diagnostic biomarkers and treatment strategies.

The question of how fungi form antifungal-tolerant or persistent subpopulations from single cells is intriguing to me. However, most previous techniques for studying antifungal tolerance or persistence, such as RNAseq, are at the population level, and a cell population has to heterogeneously express certain genes to respond and adapt to dramatic changes in environmental conditions (20). Therefore, this type of analysis has an inherent disadvantage: if there are both drug-sensitive and drug-tolerant cells in the heterogeneous population, the average effect caused by such ensemble methods will be biased, and features of subpopulations will be buried in the whole population of cells. To precisely and accurately dissect the heterogeneity of such complex microbial systems and provide reasonable explanations of the biological phenomena involved, many research teams, including my own, are now developing fungal single-cell sequencing methods (21–23). I am excited about the future of finding biomarkers of antifungal-tolerant or persistent subpopulations and developing clinical diagnostics to help us manage this cunning drug adaptation strategy of fungal pathogens.
